# Antimicrobial Resistance of *Escherichia coli* O26, O103, O111, O128, and O145 from Animals and Humans

**DOI:** 10.3201/eid0812.020770

**Published:** 2002-12

**Authors:** Carl M. Schroeder, Jianghong Meng, Shaohua Zhao, Chitrita DebRoy, Jocelyn Torcolini, Cuiwei Zhao, Patrick F. McDermott, David D. Wagner, Robert D. Walker, David G. White

**Affiliations:** * University of Maryland, College Park, Maryland, USA; † U.S. Food and Drug Administration, Laurel, Maryland, USA; ‡ The Pennsylvania State University, University Park, Pennsylvania, USA

**Keywords:** Escherichia coli, E. coli, antimicrobial resistance, Shiga toxin, STEC

## Abstract

Susceptibilities to fourteen antimicrobial agents important in clinical medicine and agriculture were determined for 752 *Escherichia coli* isolates of serotypes O26, O103, O111, O128, and O145. Strains of these serotypes may cause urinary tract and enteric infections in humans and have been implicated in infections with Shiga toxin–producing *E. coli* (STEC). Approximately 50% of the 137 isolates from humans were resistant to ampicillin, sulfamethoxazole, cephalothin, tetracycline, or streptomycin, and approximately 25% were resistant to chloramphenicol, trimethoprim-sulfamethoxazole, or amoxicillin-clavulanic acid. Approximately 50% of the 534 isolates from food animals were resistant to sulfamethoxazole, tetracycline, or streptomycin. Of 195 isolates with STEC-related virulence genes, approximately 40% were resistant to sulfamethoxazole, tetracycline, or streptomycin. Findings from this study suggest antimicrobial resistance is widespread among *E. coli* O26, O103, O111, O128, and O145 inhabiting humans and food animals.

The emergence and dissemination of antimicrobial resistance in bacteria has been well documented as a serious problem worldwide (1). Selective pressure favoring antimicrobial-resistant phenotypes is applied whenever antimicrobials are used, including treating disease in clinical medicine and preventing disease and promoting growth in animal husbandry. As a consequence, antimicrobial-resistant bacteria are selected for, thereby posing a critical public health threat in that antimicrobial treatment efficacy may be reduced.

Escherichia coli are facultative anaerobes in the normal intestinal flora of humans and animals (2,3); however, pathogenic strains of these bacteria are an important cause of bacterial infections. In humans, these strains are the foremost cause of urinary tract infections (4), as well as a major cause of neonatal meningitis (5), nosocomial septicemia, and surgical site infections (6). Infection with Shiga toxin–producing E. coli (STEC) may also result in complications including thrombocytopenic purpura, severe hemorrhagic colitis, and hemolytic uremic syndrome (7). While therapeutic options vary depending on the type of infection, antimicrobials including trimethoprim-sulfamethoxazole, fluoroquinolones, and third-generation cephalosporins are generally recommended for treating infections caused by E. coli other than STEC (6). In contrast, because these antimicrobials may increase levels of free Shiga toxin in vivo, thus facilitating disease progression, the usefulness of antimicrobials in treating STEC infection remains less clear (6,8).

Recent reports have suggested the use of tetracyclines, sulfa drugs, cephalosporins, and penicillins to be a major factor in the emergence and dissemination of antimicrobial-resistant E. coli (9–14). However, a relative paucity of information exists regarding antimicrobial resistance in E. coli from nonhospital sources, especially those from animal sources. In this study, antimicrobial susceptibility profiles were determined for E. coli isolates of serotypes O26, O103, O111, O128, and O145. Strains of these serotypes may cause urinary tract and enteric infections in humans and have been implicated in infections with STEC (15–19). The isolates were originally gathered from diverse sources, including food animals, companion animals (i.e. dogs, cats, and rabbits), and humans. Our primary objective was to characterize the extent of antimicrobial resistance in these E. coli serotypes from agricultural and clinical settings.

## Methods

### Bacterial Strains

We included 752 E. coli isolates from the collection of The Pennsylvania State University’s E. coli Reference Center in the study (Table 1); this center provides characterization of E. coli isolates submitted from outside sources. Sixty-eight isolates from humans were submitted to the E. coli Reference Center from 9 U.S. states, 45 from Saudi Arabia, 13 from Argentina, 4 from Canada, 3 from Mexico, 3 from Zambia, and 1 from Singapore. Two hundred forty-eight isolates from cattle were submitted from Michigan, 56 from Iowa, 33 from Pennsylvania, 65 from 13 other U.S. states, and 2 from Canada. Fifty-one isolates from turkeys were submitted from 13 U.S. states. Forty-five isolates from chickens were submitted from 10 U.S. states, 2 from Canada, and 2 from India. Twenty-two isolates from swine were submitted from 7 U.S. states, 3 from South Korea, and 1 from India. Seventy-four isolates from nonfood animals were submitted from 20 U.S. states, 5 from Paraguay, and 2 from Hungary. We classified nonfood animals as those not commonly used in food production, including rabbits (19 E. coli isolates), hamsters (8 isolates), deer (7 isolates), horses (7 isolates), dogs (7 isolates), alpacas (5 isolates), okapi (4 isolates), parrots (4 isolates), sheep (4 isolates), antelope (4 isolates), mice (3 isolates), seagulls (2 isolates), a cat (1 isolate), a goat (1 isolate), a llama (1 isolate), a marmoset (1 isolate), a mink (1 isolate), a rat (1 isolate), and a turtle (1 isolate).

**Table 1:** Source of isolation, genotype, serotype, and year of isolation of *Escherichia coli* isolates

	Genotype			Serotype					Year				
Source	No. isolates	STEC^a^	Other *E. coli*	O26	O103	O111	O128	O145	1976-1980	1981-1985	1986-1990	1991-1995	1996-2000
Human	137	37	100	19	23	37	53	5	0	19	4	87	27
Cow	408	140	268	230	65	60	18	35	15	16	37	60	280
Turkey	51	3	48	3	9	38	0	1	0	3	28	2	18
Chicken	49	0	49	14	21	10	3	1	5	5	21	5	13
Pig	26	3	23	9	7	2	7	1	10	6	6	1	3
Nonfood animals	81	12	69	11	43	0	13	14	0	19	30	5	27
Totals	752	195	557	286	168	147	94	57	30	68	126	160	368

^a^STEC, Shiga toxin–producing *E. coli*, determined by the presence of *stx1* and/or *stx2*.

### Antimicrobial Susceptibility Testing

Antimicrobial susceptibility testing of all isolates was done with broth microdilution using the PASCO MIC/ID system (Becton, Dickinson and Company, Sparks, MD). Testing was done according to manufacturer’s instructions and according to guidelines developed by the National Committee for Clinical Laboratory Standards (NCCLS) (20). Tested antimicrobials, dilution ranges, and resistance breakpoints are listed in Table 2. Ceftiofur- and cefoxitin-resistant isolates were further examined for production of extended-spectrum--lactamases (ESBLs) with disk diffusion according to NCCLS standards (21).

**Table 2:** Class, dilution range, and resistant breakpoints of tested antimicrobials^a^

Class or antimicrobial	Dilution range tested (µg/mL)	NCCLS resistance breakpoint (µg/mL)
**Cephalosporins**		
Cefoxitin	1–32	32
Ceftiofur	1–16	8^b^
Ceftriaxone	0.06–64	64
Cephalothin	1–32	32
**Penicillins**		
Amoxicillin-clavulanic acid	0.25/0.12–32/16	32/16
Ampicillin	0.25–32	32
**Sulfonamides and potentiated sulfonamides**		
Sulfamethoxazole	32–512	512
Trimethoprim-sulfamethoxazole	0.06/1.19–4/76	4/76
**Phenicols**		
Chloramphenicol	1–32	32
**Quinolones and fluoroquinolones**		
Ciprofloxacin	0.004–8	4
Nalidixic acid	2–256	32
**Aminoglycosides**		
Gentamicin	0.25–16	16
Streptomycin	1–256	64^b^
**Tetracycline**	1–16	16

^a^NCCLS, National Committee for Clinical Laboratory Standards. Antimicrobial susceptibility testing was performed according to NCCLS standards (20). *Escherichia coli* (ATCC 25922 and ATCC 35218), *Enterococcus faecalis* (ATCC 51299), and *Pseudomonas* *aeurigonosa* (ATCC 27853) were used as quality controls. ^b^NCCLS breakpoint not established for *E. coli*.

### Detection of Virulence Genes

Isolates were grown at 37°C overnight on veal infusion agar (Becton, Dickinson and Company). A loopful of culture was resuspended in 200 µL of distilled water, incubated at 99°C for 15 min, and centrifuged at 12,000 x g for 2 min. The supernatant was used as a template for amplification of Shiga toxin genes (stx1 and stx2), the intimin gene (eae), and the enterohemolysin A gene (hlyA) through multiplex polymerase chain reaction (PCR) (22). Primers described by Witham et al. (23) and Paton (24) were used for amplification of stx1 and stx2, respectively; those described by Gannon et al. (25) were used for amplification of eae; and those described by Fagan et al. (26) were used for amplification of hlyA. Each 11-µL PCR contained 37.5 ng stx1 primers, 15 ng stx2 primers, 15 ng eae primers, 75 ng hlyA primers, 0.18mM each deoxyribonucleotide, 4.0mM MgCl2, 50mM Tris-HCl (pH 8.3), 275 ng bovine serum albumin, 2% sucrose, 0.1mM Cresol Red (Idaho Technology, Inc., Salt Lake City, UT), and 0.4 U Taq DNA polymerase (PGC Scientifics Corp., Gaithersburg, MD). Reaction contents were cycled as described (11) after which products were electrophoresed in 1% agarose gels at 200 V for 30 min and visualized under ultraviolet light. E. coli O157:H7 (ATCC 43895) was the positive control for all reactions.

## Results

### Antimicrobial Resistance Compared to Isolation Source

**Figure 1:**
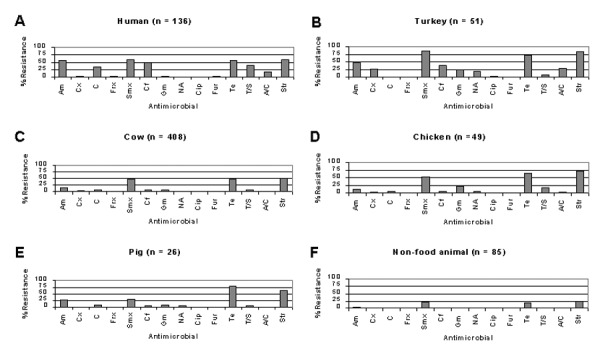
Comparison of antimicrobial resistance frequencies for Escherichia coli isolates from different sources. Am, ampicillin; Cx, cefoxitin; C, chloramphenicol; Frx, ceftriaxone; Smx, sulfamethoxazole; Cf, cephalothin; Gm, gentamicin; NA, nalidixic acid; Cip, ciprofloxacin; Fur, ceftiofur; Te, tetracycline; T/S, trimethoprim-sulfamethoxazole; A/C, amoxicillin-clavulanic acid; Str, streptomycin.

Of the isolates in this study, the highest frequencies of antimicrobial-resistant phenotypes were observed for E. coli isolates from humans and turkeys (Figure 1). Fifty-nine percent of isolates from humans were resistant to sulfamethoxazole, 59% to streptomycin, 56% to ampicillin, 56% to tetracycline, 50% to cephalothin, 38% to trimethoprim-sulfamethoxazole, 34% to chloramphenicol, and 18% to amoxicillin-clavulanic acid (Figure 1A). Eighty-four percent of isolates from turkeys were resistant to sulfamethoxazole, followed by 82% to streptomycin, 71% to tetracycline, 49% to ampicillin, 39% to cephalothin, 28% to amoxicillin-clavulanic acid, 24% to gentamicin, and 20% to nalidixic acid (Figure 1B). Nalidixic acid-resistant isolates from turkeys were found to have ciprofloxacin MICs ranging from 0.12 to >8 µg/mL, whereas each of the nalidixic acid-susceptible isolates from these animals were found to have ciprofloxacin MICs of 0.03 µg/mL or less (data not shown).

Resistance profiles among isolates from cattle, chicken, and swine were largely similar to each other (Figure 1). Fifty percent of isolates from cattle were resistant to streptomycin, followed by 47% to tetracycline, 46% to sulfamethoxazole, and 15% to ampicillin (Figure 1C). Seventy-one percent of isolates from chickens were resistant to streptomycin, followed by 63% to tetracycline, 53% to sulfamethoxazole, 20% to gentamicin, 16% to trimethoprim-sulfamethoxazole, and 12% to ampicillin (Figure 1D). Eighty-one percent of isolates from swine were resistant to tetracycline, followed by 62% to streptomycin, 31% to sulfamethoxazole, and 27% to ampicillin (Figure 1E).

Resistance frequencies were lowest for isolates from nonfood animals (Figure 1F); however, 25% were resistant to streptomycin, 20% to sulfamethoxazole, and 18% to tetracycline. Of these streptomycin-, sulfamethoxazole-, and tetracycline-resistant isolates, 76%, 82%, and 67%, respectively, were from companion animals.

Of 174 isolates resistant to ampicillin, 73% were resistant to streptomycin and tetracycline. Of 23 isolates resistant to cefoxitin, 91% were resistant to amoxicillin-clavulanic acid. Each of the five ceftiofur-resistant isolates was resistant to cefoxitin and amoxicillin-clavulanic acid. Based on NCCLS interpretive criteria for confirmatory ESBL testing (21), none of the ceftiofur- or cefoxitin-resistant isolates exhibited phenotypes consistent with ESBL production.

### Presence of Virulence Genes and Antimicrobial Resistance in STEC

Based on the presence of stx1 and stx2, 26% of the isolates were characterized as STEC. Of these, 89% contained stx1 only, 2% contained stx2 only, and 9% contained both. Eighty-one percent of STEC possessed eae and hlyA, 7% eae only, and 7% hlyA only. Of isolates that were not characterized as STEC, 34% possessed eae and hlyA, 2% eae only, and 24% hlyA only (data not shown).

The highest frequency of STEC was among isolates from cattle, in which 34% were characterized as STEC, followed by 27% of isolates from humans, 14% of isolates from nonfood animals, 12% of isolates from swine, and 6% of isolates from turkeys. None of the isolates from chickens were characterized as STEC.

**Figure 2:**
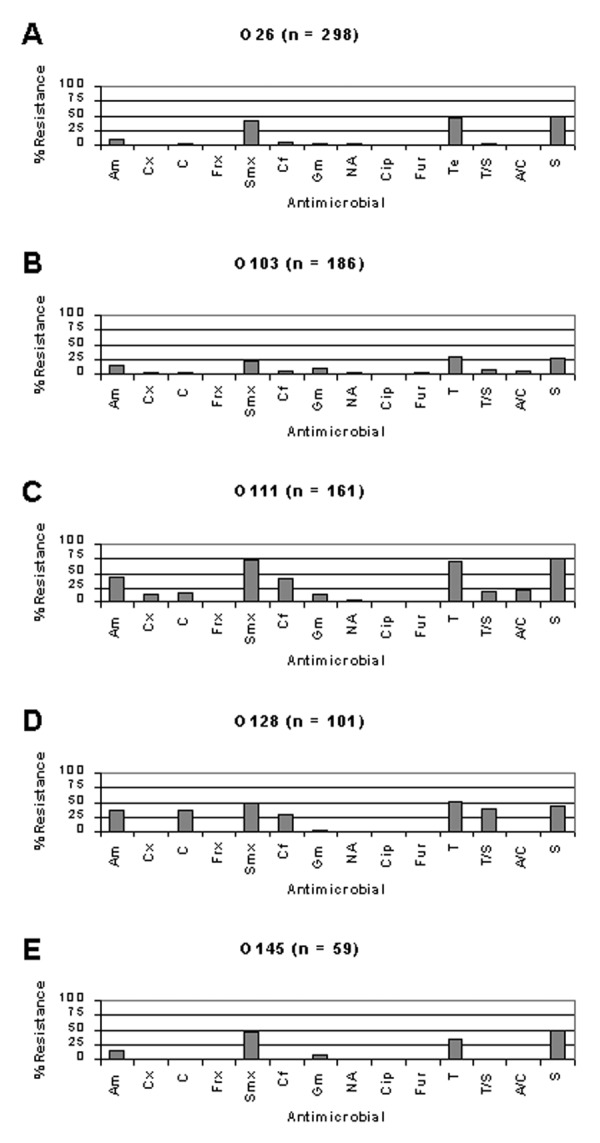
Comparison of antimicrobial resistance frequencies between Shiga toxin–producing Escherichia coli (STEC) and other E. coli. Of isolates from cattle, resistance frequencies were similar between STEC and other E. coli (A). In contrast, of isolates from humans, resistance frequencies were generally lower for STEC compared with other E. coli (B). Am, ampicillin; Cx, cefoxitin; C, chloramphenicol; Frx, ceftriaxone; Smx, sulfamethoxazole; Cf, cephalothin; Gm, gentamicin; NA, nalidixic acid; Cip, ciprofloxacin; Fur, ceftiofur; Te, tetracycline; T/S, trimethoprim-sulfamethoxazole; A/C, amoxicillin-clavulanic acid; Str, streptomycin.

Of E. coli isolates from cattle, resistance frequencies were generally similar between STEC and other E. coli, respectively, with the exception of ampicillin (26% vs. 8%), chloramphenicol (14% vs. 4%), cephalothin (14% vs. 3%), and trimethoprim-sulfamethoxazole (11% vs. 2%), in which resistance frequencies were noticeably higher (Figure 2A). In contrast, of isolates from humans, resistance frequencies were generally lower among STEC isolates compared with other E. coli (Figure 2B). Specifically, resistance frequencies were lower in STEC compared with other E. coli, respectively, for ampicillin (14% vs. 71%), chloramphenicol (5% vs. 44%), sulfamethoxazole (30% vs. 68%), cephalothin (11% vs. 64%), tetracycline (32% vs. 63%), trimethoprim-sulfamethoxazole (8% vs. 48%), amoxicillin-clavulanic acid (5% vs. 22%), and streptomycin (32% vs. 67%).

## Discussion

Of the 752 E. coli isolates characterized in this study, approximately half displayed resistance to one or more antimicrobials, including penicillins, sulfonamides, cephalosporins, tetracyclines, and aminoglycosides. These data are in accord with multiple previous studies suggesting use of these drugs has been a key factor in the emergence of antimicrobial-resistant E. coli (10-13,27,28). In addition, several other findings from this study are noteworthy in terms of their public health importance.

Approximately 40% of E. coli from humans was resistant to trimethoprim-sulfamethoxazole. Because this drug combination is recommended for treating a range of human infections, including complicated urinary tract infections, acute uncomplicated cystitis, and pyelonephritis (6), E. coli isolates should be monitored for further dissemination of trimethoprim-sulfamethoxazole resistance. Virtually all trimethoprim-sulfamethoxazole-resistant isolates from this study, however, were susceptible to ciprofloxacin and ceftriaxone, both of which are important antimicrobials for treating infections caused by trimethoprim-sulfamethoxazole-resistant E. coli.

Ceftiofur is the sole extended-spectrum cephalosporin approved for use in food animals in the United States, and it is not approved for use in human clinical medicine (29). The observation, therefore, that two isolates from humans displayed resistance to ceftiofur suggests the transfer of resistant E. coli from food animals to humans (28,30,31). However, because these two isolates also displayed resistance or decreased susceptibility to other -lactam antimicrobials, including ampicillin, amoxicillin-clavulanic acid, cephalothin, cefoxitin, and ceftriaxone, ceftiofur-resistance in these isolates might have been because of -lactam use in clinical medicine. Similarly, the relatively high number of cefoxitin-resistant isolates from turkeys compared to those from other sources may be attributable to -lactam antimicrobial use in turkey production. While, based on confirmatory tests, none of the ceftiofur- or cefoxitin-resistant isolates identified in this study yielded phenotypes consistent with ESBL production, these isolates may have produced plasmid-mediated AmpC-like -lactamases, similar to those described for other E. coli and Salmonella isolated from food animals (28–30). Consequently, work is ongoing to further characterize the genetic basis of -lactam resistance in these isolates.

The observation that 20% of E. coli isolates from turkeys were resistant to nalidixic acid (concomitant with increased MICs for ciprofloxacin) is important considering fluoroquinolones are used to treat a range of E. coli infections in humans (6). This finding, similar to those of previous reports (14,32,33), may be largely attributable to fluoroquinolone use in turkeys. The impact of fluoroquinolones such as enrofloxacin in turkey production on the emergence of quinolone- and fluoroquinolone-resistant bacteria should continue to be monitored.

Virtually all E. coli isolates from nonfood animals were susceptible to each of the antimicrobials tested. Notable exceptions, however, were isolates from dogs, cats, and rabbits. While these data yield preliminary evidence suggesting companion animals may be an important reservoir of antimicrobial-resistant E. coli of these serotypes, additional studies are required to more clearly define the impact of antimicrobial use in companion animal medicine on the emergence of antimicrobial-resistant E. coli.

STEC-associated virulence genes, including stx1, stx2, eae, and hlyA, were detected primarily in isolates from humans and cattle. Differences in pathogenicity of STEC for these two hosts may explain why STEC from humans had a higher frequency of antimicrobial resistance compared to STEC from cattle. Specifically, because in human clinical medicine antimicrobials are likely used less often to treat STEC infections compared with other E. coli infections (6,8), frequencies of antimicrobial resistance for STEC were generally lower than those for other E. coli from humans. In contrast to humans, cattle are asymptomatic carriers of STEC (34); thus the decision to use antimicrobials in cattle production does not depend upon whether or not these bacteria are present. Accordingly, antimicrobial resistance frequencies of STEC and other E. coli from cattle were largely similar to each other.

The multiple antimicrobial-resistant phenotypes observed in this study may have resulted from the spread of mobile genetic elements. For example, the observation that nearly 75% of ampicillin-resistant E. coli isolates were also resistant to streptomycin and tetracycline suggests resistance genes for these drugs are linked on plasmids. Moreover, the widespread resistance to sulfamethoxazole implies the presence of class I integrons, which are also important in conferring resistance to multiple antimicrobials (35). Research is continuing to further characterize sulfamethoxazole-resistant E. coli for the presence of these mobile genetic elements.

Because the isolates from this study were to a large extent unevenly distributed as to source of isolation versus year of isolation, analyzing resistance trends over time was not possible. Likewise, meaningful analysis of antimicrobial resistance in relation to geographic origin or to serotype was not possible. Long-term prospective studies examining isolates from defined geographic locales are required to more precisely detect temporal and spatial differences in antimicrobial resistance in strains of E. coli.

Emergence and dissemination of antimicrobial resistance in E. coli strains of serotypes O26, O103, O111, O128, and O145 may complicate treatment of certain urinary tract and enteric infections in humans and animals. Data from this study did not demonstrate a steadfast link between antimicrobial use in any particular venue and development of antimicrobial resistance among these E. coli isolates. The data did, however, suggest that antimicrobial use in clinical medicine and in agriculture was important in the selection of antimicrobial-resistant phenotypes. Continued surveillance of E. coli collected from agricultural and clinical settings, including the food production continuum, is merited to identify emerging antimicrobial-resistant phenotypes.
