# Correction, Vol. 8, No. 12

**DOI:** 10.3201/eid0901.020353

**Published:** 2003-01

In the article, “Antimicrobial Resistance of *Escherichia coli* O26, O103, O111, O128, and O145 from Animals and Humans” by Carl M. Schroeder et al., errors occurred in Figure 2 on page 1412. The corrected figure appears online at http://www.cdc.gov/ncidod/eid/vol8no12/02-0070.htm.

We regret any confusion these errors may have caused.

